# Achieving 100% amplitude modulation depth in the terahertz range with graphene-based tuneable capacitance metamaterials

**DOI:** 10.1038/s41377-025-01945-4

**Published:** 2025-08-04

**Authors:** Ruqiao Xia, Nikita W. Almond, Wadood Tadbier, Stephen J. Kindness, Riccardo Degl’Innocenti, Yuezhen Lu, Abbie Lowe, Ben Ramsay, Lukas A. Jakob, James Dann, Stephan Hofmann, Harvey E. Beere, Sergey A. Mikhailov, David A. Ritchie, Wladislaw Michailow

**Affiliations:** 1 Cavendish Laboratory, University of Cambridge, Cambridge, UK; 2 Department of Engineering, University of Cambridge, Cambridge, UK; 3 School of Electronic Engineering and Computer Science, Queen Mary University of London, London, UK; 4 Department of Electronic & Electrical Engineering, Faculty of Engineering Sciences, University College London, London, UK; 5 Institute of Physics, University of Augsburg, Augsburg, Germany; 6 Centre for Integrative Semiconductor Materials and Department of Physics, Swansea University, Swansea, UK

**Keywords:** Metamaterials, Photonic devices, Terahertz optics, Optical properties and devices

## Abstract

Effective control of terahertz radiation requires fast and efficient modulators with a large modulation depth—a challenge that is often tackled by using metamaterials. Metamaterial-based active modulators can be created by placing graphene as a tuneable element shunting regions of high electric field confinement in metamaterials. However, in this common approach, the graphene is used as a variable resistor, and the modulation is achieved by resistive damping of the resonance. In combination with the finite conductivity of graphene due to its gapless nature, achieving 100% modulation depth using this approach remains challenging. Here, we embed nanoscale graphene capacitors within the gaps of the metamaterial resonators, and thus switch from a resistive damping to a capacitive tuning of the resonance. We further expand the optical modulation range by device excitation from its substrate side. As a result, we demonstrate terahertz modulators with over four orders of magnitude modulation depth (45.7 dB at 1.68 THz and 40.1 dB at 2.15 THz), and a reconfiguration speed of 30 MHz. These tuneable capacitance modulators are electrically controlled solid-state devices enabling unity modulation with graphene conductivities below 0.7 mS. The demonstrated approach can be applied to enhance modulation performance of any metamaterial-based modulator with a 2D electron gas. Our results open up new frontiers in the area of terahertz communications, real-time imaging, and wave-optical analogue computing.

## Abstract (web summary)

By switching from a variable resistance to a tunable capacitance modulation principle using nanoscale lateral capacitors and leveraging substrate-side reflection, we achieve 100% amplitude modulation in graphene-based metamaterial terahertz modulators.

## Graphical abstract

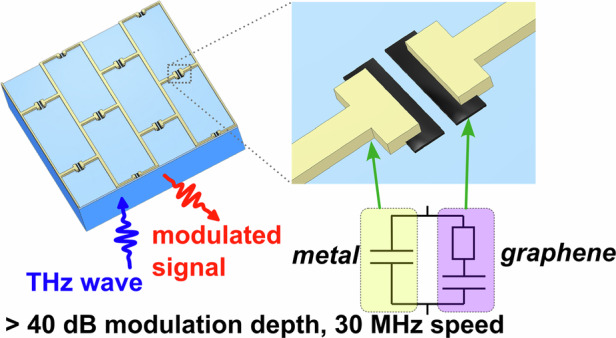

## Introduction

The ability to manipulate the properties of electromagnetic waves, such as the amplitude^1^, phase^2^, or polarisation^3^ of the electric field, is essential in a variety of applications. While modulation technology is rather mature in the near-infrared and microwave ranges, the search for fast and efficient modulation methods in the terahertz (THz) range is still ongoing.

Direct electronic modulation, used in the microwave regions and below, cannot be easily adapted to the sources used in the THz range. At the same time, established modulation technologies used in the near-optical 1.55 µm telecoms range, such as using silicon waveguide photonics^4–7^, or in the 8–10 µm mid-infrared range^8–10^, cannot be easily transferred to the terahertz range, due to reasons including a lack of suitable waveguide structures, much larger wavelengths, and a resulting increase in device size and thus capacitance that costs orders of magnitude in modulation speed. In the THz range, available methods of amplitude modulation using electrical control include micro-electromechanical systems (MEMS)^11,12^, devices employing vanadium oxide (VO_2_) as a material undergoing a thermally activated phase change^13,14^, liquid crystals^15–17^, and liquid ion-gating of graphene^18–20^. While these methods offer a large modulation depth and low insertion loss, applications requiring speeds above the MHz range demand solid state devices that do not rely on mechanical or thermal changes. This demand can be met with active metamaterials, which consist of artificially engineered subwavelength structures with electrically tuneable elements^2,21,22^. These elements allow the optical properties of the metamaterials to be dynamically adjusted^2,22–29^; detailed reviews of the performance of modulators are presented in e.g. refs. ^30,31^.

In active metamaterial modulators, a thin conductive layer is typically placed under the capacitive gaps within the structure where the electric field has its largest confinement^1,30,32–34^. Graphene is a promising candidate for this tuneable element, thanks to its large, electrically controllable conductivity, which can be varied by applying a gate voltage between the graphene layer and a back gate^35^. Without graphene, the structure represents an effective inductor–capacitor (LC) circuit, with a transmission or reflection spectrum having a resonant shape with a resonance frequency determined by geometrical parameters of the metamaterial. Placing graphene under the capacitor element leads to a shunting of the capacitor by a resistor with a variable and controllable resistance. This enables control of the transmission/reflection spectra of the structure and thus modulation of the intensity of the transmitted/reflected THz radiation. The advantage of metamaterial modulators is their potential for high-speed operation, with speeds beyond 10 MHz and into the GHz range^34^. However, the shorting of the capacitor with a resistor represents a damping of the oscillating circuit, which has the disadvantage of limiting the attainable modulation efficiency and range.

A second issue pertinent to graphene in particular is its finite conductivity at the Dirac point, which does not allow graphene-based channels to be fully depleted. This lower limit of the conductivity directly translates into a restriction on the modulation depth of single-layer THz modulators operating in transmission.

As a result, with metamaterial-based devices operating in the THz range, the modulation depth, defined as

1 $$h=\frac{{A}_{{\rm{Max}}}-{A}_{{\rm{Min}}}}{{A}_{{\rm{Max}}}}$$

has been rather limited, with *h* < 10 dB for free-standing devices capable of operating at such speeds^1,22,34,36,37^. Here ${A}_{{\rm{Max}}}$ and ${A}_{{\rm{Min}}}$ are the maximum and minimum values of an optical parameter that can be electrically controlled, such as the electric field amplitude of the reflected or transmitted wave.

Many approaches to increase the modulation depth have been explored. They include quarter wave resonance cavities^18,38^, multilayer graphene structures^19^, large Fermi-level tuning in graphene using ion gels^18–20^, as well as reflection geometries similar to the Salisbury screen exploiting critical coupling^20^, Brewster-angle devices^39^, and total internal reflection geometries^40^. Semiconductor-based capacitive/inductive detuning of metamaterials has been shown using silicon-on-sapphire, but relied on ultrafast, pulsed lasers to induce the required very large conductivities^41^. The challenge lies in the fact that in the race for a large modulation depth, one has to sacrifice speed (e.g. the kHz-speeds of liquid ion gating), and vice versa; both can be achieved simultaneously but require pulsed lasers, etc. Thus, it is difficult to realise a modulator that can combine large modulation depth with high speeds (>10 MHz), while relying on easily accessible materials and solid-state components that can be electrically controlled—although that is exactly the type of component needed in many practical terahertz applications.

In our work, we propose and demonstrate two solutions to this challenge. Firstly, instead of shunting the metamaterial gaps with graphene and exploiting its resistive dampening, we utilise graphene as a tuneable capacitor by designing the graphene areas as patches protruding from the nanoscale capacitor metal plates. Secondly, we exploit destructive interference of Fresnel reflection components from the substrate and the metamaterial, by exciting the modulator from its rear side, i.e. the substrate, to zero out the reflection and achieve unity modulation. In combination, these approaches allow us to demonstrate electrically driven, single-layer, all-solid-state, graphene-metal metamaterial modulators operating in the THz range at room temperature. The modulators exhibit modulation depths in excess of four orders of magnitude in intensity, with a reconfiguration speed of 30 MHz.

## Results

### Device structure and operating principle

**Fig. 1:**
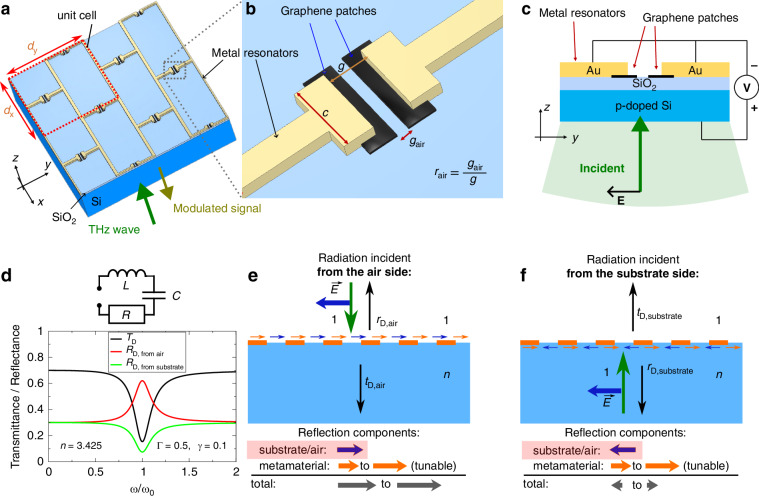
**Schematic of the brickwork antenna tuneable capacitance modulator**. **a** 3D view of the device area, the unit cell is indicated by a red dashed box; **b** an enlarged view of a metamaterial capacitor with the air gap in the graphene patch; and **c** a cross-sectional view of the device with the back-gate voltage source indicated. **d** Optical response of a “basic” L-C-R metamaterial structure: transmittance (black curve), reflectance of radiation incident from the air side (red curve) and from the substrate side (green curve). The curves are plotted using Eqs. (2)–(6) with *d*_*x*_ = *d*_*y*_, *n* = 3.425, Γ/*ω*_0_ = 0.5, and *γ*/*ω*_0_ = 0.1. **e**, **f** Reflection from a metamaterial on a substrate with refractive index *n*: comparison between the excitation **e** from the top side and **f** from the back side. Vertical arrows show the direction of the electromagnetic wave vector; the horizontal arrows indicate the electric field direction. The coloured arrows indicate the direction of the reflected electric field from the substrate–air interface (blue) and from the metamaterial (orange)

Our device consists of a metamaterial structure having the shape of a periodic brickwork antenna array, Fig. 1a. The structure lies on a dielectric substrate and can be irradiated by a *y*-polarised electromagnetic wave with the frequency *ω*, normally incident from the top (air) or bottom (dielectric substrate, Fig. 1c) side. The metamaterial structure is designed in such a way that the transmittance *T*(*ω*) and reflectance *R*(*ω*) of the electromagnetic wave have a resonance in a terahertz range. The periods of the structure in the *x* and *y* directions, *d*_*x*_ and *d*_*y*_, are small compared to the wavelength of radiation in free space *λ*.

To quantitatively describe the *T*(*ω*) and *R*(*ω*) curves, one should solve Maxwell’s equations in the system “air–thin metamaterial layer–dielectric”. The air and dielectric can be described as uniform media with the refractive indices 1 and *n*, respectively. Since *d*_*x*_ and *d*_*y*_ are smaller than the radiation wavelength, we can, like in conventional macroscopic electrodynamics, describe the metamaterial layer as a thin, two-dimensional (2D) layer with an effective 2D conductivity *σ*(*ω*). Then the solution of Maxwell’s equations gives

2 $${T}_{{\rm{D}},\;{\rm{from}}\; {\rm{air}}}={T}_{{\rm{D}},\;{\rm{from}}\; {\rm{substrate}}}=n{\left|\frac{2}{n+1+{Z}_{0}\sigma (\omega )}\right|}^{2}$$

3 $${r}_{{\rm{D}},\;{\rm{from}}\; {\rm{air}}}=-\frac{n-1+{Z}_{0}\sigma \left(\omega \right)}{n+1+{Z}_{0}\sigma \left(\omega \right)},{R}_{{\rm{D}},\;{\rm{from}}\; {\rm{air}}}={|{r}_{{\rm{D}},\;{\rm{from}}\; {\rm{air}}}|}^{2}$$

4 $${r}_{{\rm{D}},\;{\rm{from}}\; {\rm{substrate}}}=\frac{n-1-{Z}_{0}\sigma (\omega )}{n+1+{Z}_{0}\sigma (\omega )},{R}_{{\rm{D}},\;{\rm{from}}\; {\rm{substrate}}}{{={|r}}_{{\rm{D}},\;{\rm{from}}\; {\rm{substrate}}}|}^{2}$$

where *Z*_0_ is the impedance of free space (≈376 Ohm) and the *r*_D_ quantities are the amplitude reflection coefficients in the case of incidence from the air side and from the substrate side, respectively. As seen from formulas (2)–(4), the transmittance of the metasurface “device” (subscript D) does not depend on whether the radiation is incident from the substrate or from the air side, while the reflectance does.

The effective 2D conductivity of the metamaterial layer can be calculated by averaging the AC electric current, induced in the system by the incident electromagnetic radiation, over areas large compared to the period of the metamaterial, but small compared to the radiation wavelength. Let us first consider the “basic” metamaterial structure shown in Fig. 1a, without graphene patches. If the radiation is polarised in the *y*-direction, the unit cell of the brickwork structure can be represented by an equivalent circuit, consisting of a capacitor *C*, resistance *R*, and inductance *L* connected in series, Fig. 1d. The effective 2D conductivity of the metamaterial can then be written as

5 $$\sigma \left(\omega \right)=\frac{{d}_{y}}{{d}_{x}}\frac{1}{R+i\omega L+1/i\omega C}$$

and the factor ${Z}_{0}\sigma$ assumes the form

6 $${Z}_{0}\sigma \left(\omega \right)=\frac{{d}_{y}}{{d}_{x}}\frac{i\omega \Gamma }{{\omega }_{0}^{2}-{\omega }^{2}+i\omega \gamma }$$

In Eq. (6), ${\omega }_{0}^{2}=1/{CL}$ is the resonance frequency and the quantities $\gamma =R/L$ and $\Gamma ={Z}_{0}/L$, measured in units 1/s, have the physical meaning of the dissipative ($\gamma$) and radiative ($\Gamma$) decay rates. Equations (2)–(4) together with Eq. (6) give the transmittance and reflectance spectra of a “basic” metamaterial structure. Figure 1d shows typical *T*(*ω*) and *R*(*ω*) curves for some values of the parameters $\gamma$ and $\Gamma$.

In order to modulate the intensity of the transmitted and reflected radiation, one can place conductive graphene patches in the areas under the capacitive gaps, Fig. 1a, b. By applying a gate voltage to the metallic wires and hence to the graphene patches, Fig. 1c, one can then change the graphene resistance and modulate the amplitudes of the reflected or transmitted waves^1,30,32–34^. In our work, we introduce two crucial improvements compared to the state of the art, as described in the following.

### Methods to improve modulation efficiency and depth

#### Nanoscale tuneable capacitors

**Fig. 2:**
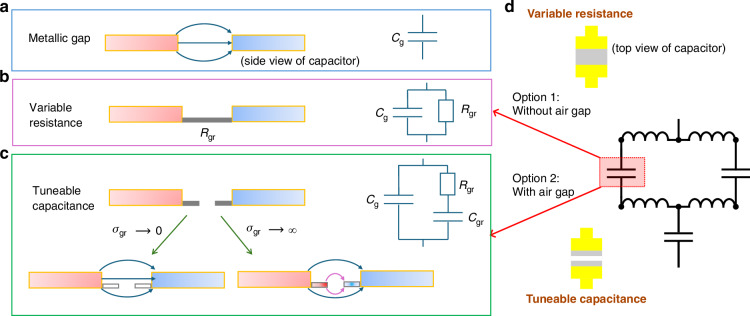
Equivalent circuit of the nanoscale capacitors and the brickwork antenna arrays. **a**–**c** The capacitors of the structure are presented in a side view: **a** pure metallic gap of the antenna structure, **b** continuous graphene patch shunting the antenna gap (variable resistance, no air gap) and **c** nanoscale graphene patches protruding from either side of the metallic capacitor (tuneable capacitance, air gap). **d** Equivalent circuit model of a full metamaterial unit cell. The model of the capacitive gap is different, depending on whether the variable resistance or tuneable capacitance approach is used. The corresponding capacitor structures are shown in a top view

In the metamaterial structure, incident radiation is confined into narrow gaps, shown in Fig. 2. Without any graphene, the gap between the antennas of the metamaterial represents a capacitor ${C}_{{\rm{g}}}$, Fig. 2a. In the majority of graphene metamaterial modulators, the graphene patches introduced to modulate the optical response are placed such that they cover the whole area under the capacitive gaps. This way, they shunt the capacitor ${C}_{{\rm{g}}}$ by a variable resistor ${R}_{{\rm{gr}}}$ in parallel with the capacitor, Fig. 2b. In this *variable resistance* method of light modulation, the resonator gap corresponds to a capacitor with variable loss. We insert an air gap in the graphene patch in the centre of the capacitor, Fig. 2c. Thus, we use the graphene patches protruding from either side of the capacitor as tuneable nanoscale capacitors instead, thereby realising a *tuneable capacitance* modulation technique. This is the first crucial improvement proposed in our work.

To illustrate how the 2D conductive layer functions as a tuneable capacitance, let us consider two limiting cases shown in Fig. 2c. At extremely low conductivity of the 2D conductive layer (i.e., ${\sigma }_{{\rm{gr}}}\to 0$), it becomes insulating, and the capacitance remains determined by the metallic antenna gap. Conversely, at very high conductivity ${\sigma }_{{\rm{gr}}}\to \infty$, the 2D layer acts as a near-perfect conductor, thus switching on a second capacitor ${C}_{{\rm{gr}}}$ in parallel with ${C}_{{\rm{g}}}$. In between these two extremes, there is an interplay of the impedances of the metallic capacitor ${C}_{{\rm{g}}}$ and of the second capacitor ${C}_{{\rm{gr}}}$, connected through the graphene resistance ${R}_{{\rm{gr}}}$, which is described by the equivalent circuit in Fig. 2c. Compared to the variable resistor tuning, the tuneable capacitor modulation enables a larger range of modulation and operation across a wider frequency window.

Quantitatively, the transmittance and reflectance spectra in these additional cases can be described by the formulas (2)–(5), in which the effective conductivity (5) should be modified according to the equivalent circuits shown in Fig. 2b, c: the term 1/(*iωC*) in the denominator of Eq. (5) should be replaced by *R*_gr_/(1 + *iωCR*_gr_) in the variable resistor case and by a similar but slightly more complicated expression, corresponding to the equivalent circuit of Fig. 2c, in the tuneable capacitor case. The equivalent circuit for a full metamaterial unit cell is shown in Fig. 2d.

#### Back excitation reflection approach

The second improvement of our work is that we modulate the light incident from the back (substrate) side and reflected from the boundary metasurface–air. In the case of top-side excitation, with the wave incident on the device from the metamaterial side, the reflection or transmission cannot be fully extinguished. However, in the case of the back-excitation reflection, it is possible to turn ${A}_{{\rm{Min}}}$ in Eq. (1) to zero. The reflectance curve can have a minimum in this case, Fig. 1(d), so that by varying the graphene resistance *R*_gr_ one can turn the reflectance to zero using a suitable choice of parameters. Mathematically, this follows from Eqs. (2)–(4): The transmission, Eq. (2), can be minimised by maximising *σ*, but will never fully reach zero. The numerator in the reflection coefficients consists of two terms: one proportional to (*n* − 1), corresponding to the Fresnel reflection due to the substrate-air refractive index mismatch, and another one with ${Z}_{0}{\rm{\sigma }}$, representing the reflection from the metamaterial. Equation (3) contains their sum, while Eq. (4) contains their difference. In the case of incidence from the air side, the numerator is proportional to (*n* – 1) + *Z*_0_*σ* and never vanishes. But the metamaterial reflection from the substrate side is proportional to the difference (*n* – 1) – *Z*_0_*σ* and can vanish. This is achieved when the complex function (*n* – 1) – *Z*_0_*σ* equals zero, which is reduced to a system of two real equations that have solutions at certain values of two parameters, angular frequency *ω* and graphene direct current (DC) conductivity *σ*_0_. This way, one can achieve zero reflectance, *A*_min_ = 0, and hence 100% modulation depth.

Physically, this difference is illustrated in Fig. 1e, f. The electric fields of the waves reflected from the metamaterial are shown by the orange arrows, and those from the Fresnel reflection due to the substrate-air refractive index mismatch are shown by the blue arrows. The total reflection at the interface between air and the metamaterial/substrate is determined by the vector sum of these two components. In the case where light is incident from the air side, the Fresnel air-substrate and the metamaterial reflection components interfere constructively, Fig. 1e. Therefore, any change in the optical properties due to the metamaterial will result in only a weak change in the total reflection amplitude. When light is incident on a surface transitioning from a medium with a lower refractive index (e.g. air) to one with a higher refractive index (e.g. substrate) (termed “top excitation”), the light wave experiences a π phase shift. Conversely, when light propagates from a medium with a higher refractive index to a lower refractive index (termed “back excitation”), no π phase shift occurs. In the case of back excitation, the Fresnel components from the metamaterial and the air–substrate contributions interfere destructively, Fig. 1e. In this case, any small change in metamaterial optical properties will be greatly enhanced, enabling 100% amplitude modulation depth.

Thus, in the case of the back-excitation reflection, it is possible to turn ${A}_{{\rm{Min}}}$ in Eq. (1) to zero achieving a full modulation of radiation. To realise this modulation approach in real devices, the reflection from the backside, i.e. the first air–silicon interface, should be suppressed. This can be done by an antireflection coating or by other methods outlined in the “Discussion” section.

### Numerical simulations of proposed device

Analytical formulas derived in the previous section provide a physical understanding of the proposed device concept. However, to make more accurate calculations corresponding to our device, we perform numerical simulations and optimisations of the brickwork antenna metamaterial structure, shown in Fig. 1a, using the RF module of COMSOL Multiphysics software (version 6.1). The capacitor width and gap length are *c* and *g*, respectively. The antenna parameters have been tailored to produce a resonant frequency at 2.4 THz without graphene present in the gaps. The resulting parameters are $g$ = 1.5 ${\rm{\mu }}{\rm{m}}$, $c$ = 3 ${\rm{\mu }}{\rm{m}}$, ${d}_{{\rm{x}}}$ = 35 µm, ${d}_{{\rm{y}}}$ = 35 µm. The width of all metallic lines in the *x*–*y*-plane is 1.2 µm. The metal thickness is 170 nm, chosen to be significantly larger than the skin depth of ~53 nm at 2.15 THz (with $\rho =2.43\times {10}^{-8}\,\Omega {\rm{m}}$; ref. ^42^). The length of the “air gap” in the graphene, i.e. the distance between the graphene lateral capacitor plates, is ${g}_{{\rm{air}}}$. The air gap ratio is defined as ${r}_{{\rm{air}}}={g}_{{\rm{air}}}/g$. We choose an exemplary air gap ratio of ${r}_{{\rm{air}}}={g}_{{\rm{air}}}/g=0.23$. The Drude model is used to simulate the behaviours of the conductive materials, with the parameters from ref. ^43^ (graphene) and refs. ^42,44^ (gold). The conductivity of the graphene patches can be continuously tuned by applying a bias voltage between the back of the p-doped silicon substrate and the metal resonator array, see Fig. 1c, which in turn changes the amplitude of the reflected wave incident from the substrate side.

**Fig. 3:**
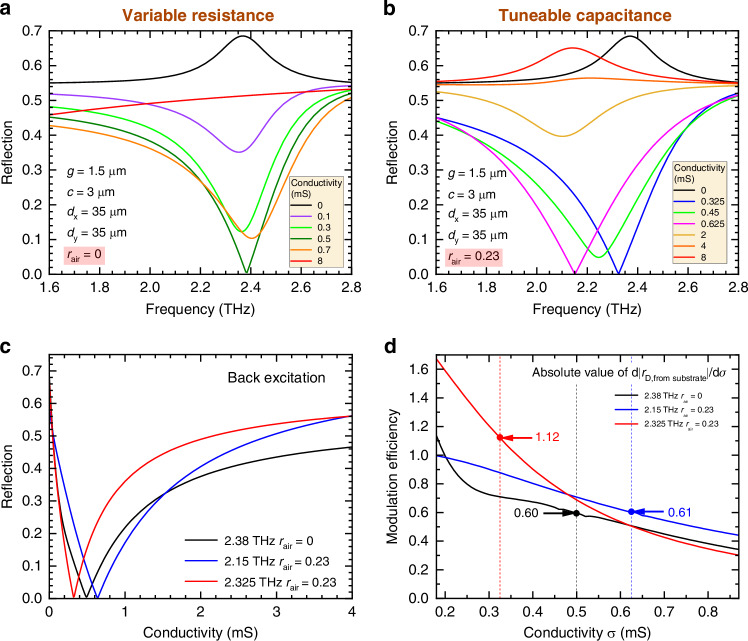
Simulated reflected electric field amplitudes for the variable resistance vs. tuneable capacitance metamaterial. The simulated structure has the parameters $g$ = 1.5 µm, $c$ = 3 µm, ${d}_{{\rm{x}}}$ = 35 µm, ${d}_{{\rm{y}}}$ = 35 µm. **a** Reflection against frequency for the variable resistance metamaterial, i.e. an air gap ratio of ${r}_{{\rm{air}}}=0$. **b** Reflection against frequency for the tuneable capacitance metamaterial with an air gap ratio of ${r}_{{\rm{air}}}=0.23$. **c** Reflection against graphene conductivity. The black curve shows the reflection at the resonant frequency of the pure metallic brickwork antenna structure for the case ${r}_{{\rm{air}}}=0$, i.e. no air gap, of 2.38 THz for the back excitation cases. The blue and red curves correspond to ${r}_{{\rm{air}}}=0.23$ and the frequency at 2.15 and 2.325 THz, where the minimum of the back excitation reflection is observed. **d** Modulation efficiency against graphene conductivity for two different types of modulation at different frequencies. The arrows in Fig. 3d indicate the modulation efficiency at the point where the reflection vanishes, and the corresponding conductivity values are indicated by the dashed lines

The simulated reflection amplitudes |${r}_{{\rm{D}},\;{\rm{from}}\; {\rm{substrate}}}$| from the brickwork antenna metamaterial structure for waves incident from the substrate against frequency at different conductivities, for the variable resistance modulator (without air gap) and tuneable capacitance modulator (with air gap), are shown in Fig. 3a, b, respectively. While the reflection can vanish in both cases, the specific behaviour of the devices is notably different.

For the variable resistance case (without air gap), as the conductivity increases, the maximum at resonance rapidly flips and turns into a minimum, which becomes deeper until the reflection fully vanishes at 2.4 THz and 0.5 mS. As the conductivity keeps increasing above 0.5 mS, the quality factor of the resonance drops, while the resonance frequency remains around 2.4 THz (within ca. 0.02 THz), until the resonance disappears due to overdamping from the graphene.

For the tuneable capacitance (with air gap) case, the reflected electric field reaches its maximum value at resonance in two cases: when the active tuneable element has zero conductivity, and when it has a very large conductivity, above ca. 4 mS. The first case corresponds to a pure metallic brickwork antenna without graphene. In the latter case, a very large conductivity effectively renders the active element (e.g. graphene) metallic, and the resonance curve approaches the shape expected from a pure metallic resonance with a reduced capacitive gap. Due to a larger capacitance in this case, this resonance occurs at a lower frequency. In between these two extremal cases, the maximum of the reflection curve at resonance is converted to a minimum. This minimum reaches full extinction, i.e. zero reflection, at two different values of the conductivity, around 0.625 and 0.325 mS, and at two different frequencies, respectively, 2.15 and 2.325 THz.

Let us now consider the difference between the case when the graphene shunts the capacitors without an air gap and the case when an air gap is introduced in the graphene patch. Figure 3c compares the absolute values of the reflection coefficients as a function of the graphene conductivity for radiation incident from the substrate side of the brickwork antenna structure. The reflection is evaluated at the frequencies where the zero in reflection is achieved and for two different cases: no air gap (black), and with an air gap at the two different frequencies. In the case of graphene shunting the capacitors (no air gap), there is only one zero of the reflection at one frequency. But in the case of the tuneable capacitance device with air gap, the reflection vanishes at two different frequencies, see the blue and red lines in Fig. 3c. This allows achieving 100% modulation depth at two different frequencies, with a large modulation depth in between, which enables frequency tunability and hence widens the operational frequency window.

**Fig. 4:**
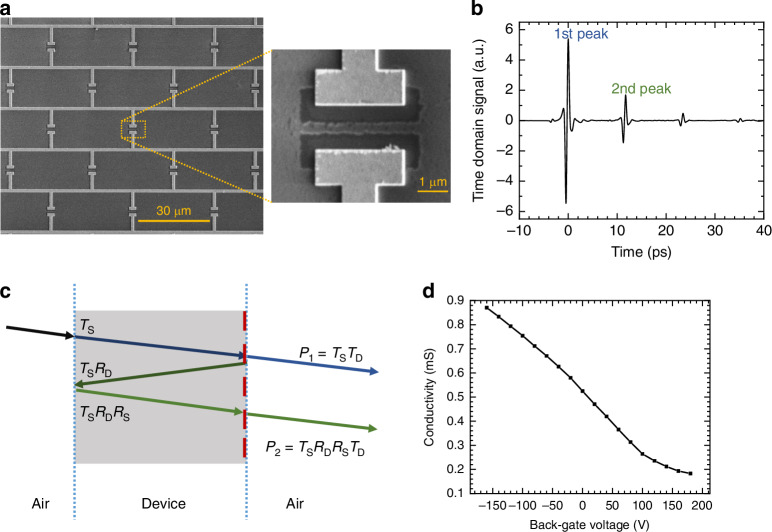
Characterisation and measurement of the fabricated tuneable capacitance modulator. **a** Scanning electron microscope (SEM) image of the brickwork antenna arrays, with a zoomed-in view of the capacitor and air gap. **b** Measured transmitted pulses in the time domain, showing the different peaks resulting from internal reflections within the device. **c** Schematic diagram of the internal reflections inside the substrate and the corresponding signals, where ${T}_{{\rm{D}}}$ and ${R}_{{\rm{D}}}$ are the transmittance and reflectance of the metamaterial, and ${T}_{{\rm{S}}}$ and ${R}_{{\rm{S}}}$ are the transmittance and reflectance of the silicon substrate. The metamaterial is indicated by the dashed red line, and the device is excited from the substrate side under normal incidence. The arrows are tilted for illustration purposes only. **d** Measured graphene conductivity as a function of the back-gate voltage applied across substrate and metal contacts

Additionally, the slopes of the two curves in Fig. 3c are also different. The slopes directly relate to the modulation efficiency, i.e., the effectiveness with which a modulator can modulate a particular property (e.g. reflection) in response to the parameter inducing the modulation. It can be quantified as the absolute value of the derivative ${\rm{d}}{{|r}}_{{\rm{D}},\;{\rm{from}}\; {\rm{substrate}}}|/{\rm{d}}\sigma$, where $\sigma$ is the external control parameter, in this case, the graphene conductivity. Figure 3d shows the modulation efficiency for the two different device types. The plot is shown as a zoom-in to the conductivity range 0.18–0.87 mS that is expected to be accessible based on DC two-contact measurements of chemical vapour deposition (CVD)-grown graphene, as shown in Fig. 4d. The red curve (with an air gap; at 2.325 THz) exceeds the black curve (without air gap) when the conductivity is below 0.6 mS, while the blue curve (with an air gap; at 2.15 THz) exceeds the black curve when the conductivity is above 0.2 mS. This demonstrates that a larger modulation efficiency can be obtained across the entire graphene conductivity range by using the tuneable capacitance approach instead of the variable resistance approach, which enables more efficient modulation for a given graphene conductivity range. The modulation efficiency corresponding to the conductivity values where the reflection vanishes is indicated by the arrows in the figure. By comparing the values at the respective zeros in reflection, one can see that the modulation efficiency can be nearly doubled (0.60 vs. 1.12).

### Device geometry and measurements

The device is fabricated on a sample with an active device area of 1.3 mm by 1.3 mm, comprising 36 × 36 unit cells and using graphene from a commercial supplier, see “Methods”. The parameters chosen for a unit cell are *c* = 3 μm, *g* = 1.5 μm, and a unit cell size of 35 μm by 35 μm. A scanning electron microscope (SEM) image of the fabricated device is shown in Fig. 4a. The device has an air gap ratio of approximately ${r}_{{\rm{air}}}$ = 0.23, as measured from SEM images; the other design parameters are displayed in Fig. 3b.

To measure the reflectance of the metamaterial at the excitation from the substrate side, we use a terahertz time-domain spectroscopy (TDS) system. The TDS measurement of the transmitted electric field shows several pulses in the time domain, see Fig. 4b. The transmittance of the metasurface is presented in the Supplementary Information, Section SI-2. From an analysis of the first and second transmitted peaks, we then extract the reflectance of the device ${R}_{{\rm{D}}}={\left|{r}_{{\rm{D}},\;{\rm{from}}\; {\rm{substrate}}}\right|}^{2}$, see Fig. 4c and “Methods”.

**Fig. 5:**
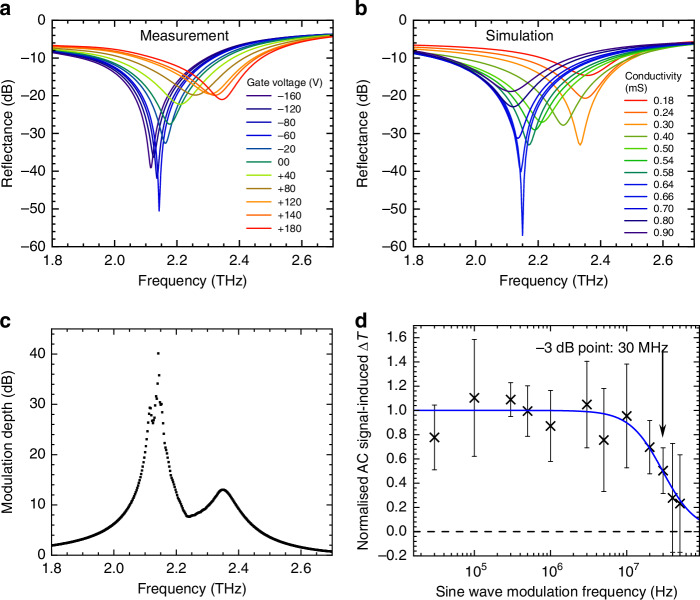
Experimental results and comparison with simulation. **a**, **b** Reflectance of the brickwork antenna tuneable capacitance metamaterial as a function of frequency: **a** measurement with gate voltage varied from −160 to 180 V, **b** numerical simulations of the structure with an air gap ratio of ${r}_{{\rm{air}}}=0.23$ for different graphene conductivities. **c** THz frequency dependence of the modulation depth. **d** Modulation speed evaluated from the difference between the DC and AC measurements taken by TDS, as a function of the driving AC sine wave frequency

The obtained reflectance ${R}_{{\rm{D}}}$ is shown in Fig. 5a for different back-gate voltages. The device achieves a modulation depth as high as 99.01% in amplitude and 99.99% in intensity, corresponding to 40.1 dB, at a frequency of 2.15 THz. The experimental data, shown on a logarithmic scale in Fig. 5a, reveals a fine structure of the minimum, which is split in two. Figure 5b shows the results of simulations of the fabricated device with the air gap ratio of ${r}_{{\rm{air}}}=0.23$ and the measured graphene conductivity range of 0.18–0.90 mS. The conductivity is estimated using two-terminal measurements on a 70 μm-by-200 μm graphene test pad close to the modulator structure, but on the same chip, having undergone the same cleanroom fabrication process, and is shown in Fig. 4d as a function of the back-gate voltage. This data was recorded simultaneously with the TDS measurement.

There is a remarkably good agreement between experimentally measured reflectance and the numerically simulated data. Both the overall trend, as well as the expected modulation ranges, are well reproduced. In the experimental data, both minima of the reflection are clearly observed and show a modulation depth well above 10 dB. At 2.15 THz, we achieve unity amplitude modulation (>40 dB modulation depth measured experimentally); at the second minimum at 2.35 THz, we achieve a modulation depth of 12 dB. We attribute this to the fact that the reflection zero at 2.35 THz appears experimentally slightly outside the experimentally attainable graphene conductivity range, owing to the fact that more precise knowledge of the gate voltage dependent relaxation time of graphene is necessary to optimally numerically simulate the device’s performance. Additionally, it should be noted that the plotted conductivities in the simulation cannot be expected to match exactly to the measured values on the 70 μm × 200 μm graphene test area, due to effects such as the contact resistance at the metal-graphene interface^45^.

To ensure reproducibility of the results, we repeated the measurements 6 times, including measurements on different days and with re-alignment of the sample position. The device has consistently demonstrated 97.65–99.41% amplitude and 99.94–100% intensity modulation depth at 2.15 THz as the back-gate voltage was changed from –160 to 180 V.

The other figure of merit of a modulator is the modulation speed, which we also measure in the TDS system. To characterise the speed of the device, we make use of an established method enabling measurements of the modulation speed with a slow detector, which relies on the difference of the temporal average of the TDS signal, observed with and without applied alternating current (AC) sine wave of varying frequency^1,33^, see “Methods” section. As seen in Fig. 5d, as the frequency of the driving signal grows, the difference $\Delta T$ between the TDS data with and without AC signal diminishes in a resistor–capacitor (RC)-type roll-off typical for metamaterial-based modulators. By fitting the data with the function $\Delta T(f)=1/[1+{(f/{f}_{3{\rm{dB}}})}^{2}]$, we extract an approximate –3 dB point of 30 MHz.

This is consistent with our estimates of the RC time constant of the device from a simple parallel plate capacitor model. The capacitance of the metasurface is estimated to be ~23.5 pF, based on the metal antenna surface area and the 300 nm silicon dioxide layer. With a device size of 1.3 mm × 1.3 mm, the resistance of the p-doped silicon substrate (*ρ* = 100 Ω⋅cm, thickness = 525 ± 25 μm) is estimated to be ~311 Ω, leading to an RC time constant of 7.31 ns and an approximate modulation speed of ~22 MHz.

### Comparison of devices with and without air gap

What would the device performance have been, if the air gap had not been introduced? To investigate this, we design and fabricate two new devices with identical metal antenna designs but different active element configurations: one with an air gap and one without. This allows a direct comparison between the tuneable capacitance and variable resistance approach. To also demonstrate the universal applicability of our approach, we choose a different operating frequency of 1.7 THz for the devices presented in this section, by expanding the size of the unit cell, and a different graphene material: fabricated in our in-house CVD machine, instead of the commercially supplied material.

The brickwork modulators are fabricated on a sample with an active device area of 1.3 mm by 1.3 mm, comprising 28 × 28 unit cells, see “Methods”. The geometrical parameters of the capacitor are kept the same, *c* = 3 μm, *g* = 1.5 μm, but the unit cell size is chosen to be larger, 45.5 μm by 45.5 μm. The air gap ratio of 0.23 for the tuneable capacitance modulator is measured using SEM images.

**Fig. 6:**
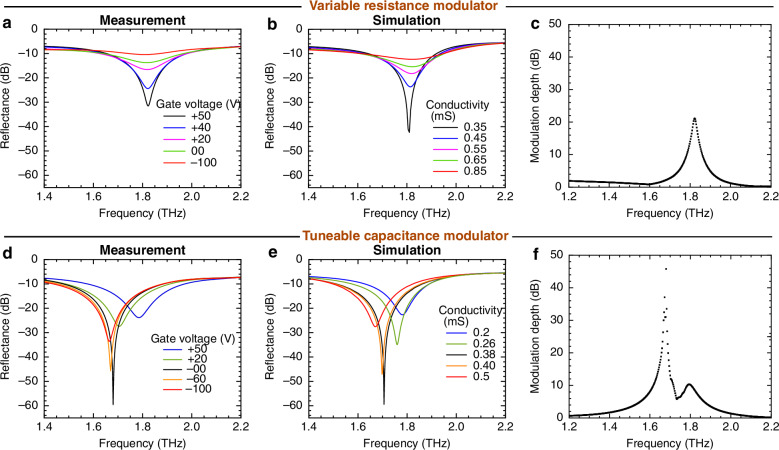
Comparison of the performance of the variable resistance and tunable capacitance modulators. Reflectance and modulation depth of the variable resistance modulator (without air gap: **a**–**c**) and tuneable capacitance modulator (with air gap, *r*_air_ = 0.23: **d**–**f**). The device structure has the parameters $g$ = 1.5 μm, $c$ = 3 μm, ${d}_{{\rm{x}}}$ = 45.5 μm, ${d}_{{\rm{y}}}$ = 45.5 μm. **a**, **d** TDS measurements of the reflectance of the brickwork antenna metamaterial as a function of frequency with gate voltage varied. **b**, **e** COMSOL simulation of the frequency-dependent reflectance of the brickwork antenna metamaterial for different conductivities. **c**, **f** Measured frequency dependence of the modulation depth

The measurements and simulations of the reflectance for both devices are plotted using the same scale on the *x* and *y* axes, enabling direct comparison of the data. The reflectance data for the no air-gap, variable-resistor device shows a modulation depth of 21 dB when varying the gate voltage from +50 V to –100 V, see Fig. 6a, b. There is no shift in resonance frequency (less than 0.02 THz) across different gate voltages. By contrast, the tuneable capacitance modulator with air gap in the graphene patches achieves amplitude modulation in excess of 99% (45.7 dB) at a centre frequency of 1.68 THz when varying the gate voltage from 0 to +50 V. Notably, even at 0 V, a large change in the reflectance enables 30 dB modulation within a 20 V range (from 0 to +20 V), demonstrating high modulation depth using moderate voltage levels. The resonant frequency of this device is around 1.8 THz when graphene is gated at low conductivity; see the blue curve in Fig. 6d, so there is an obvious resonant frequency change, larger than 0.15 THz in the measured voltage range. The transmittance measurements for both devices are provided in the supplementary document. The tuneable capacitance device achieves a 35% transmission intensity modulation depth as the gate voltage varies from +50 to –60 V, while the variable resistance device exhibits a 31% intensity modulation depth from +50 to –100 V in transmission.

## Discussion

In Table 1, we give some examples illustrating the performance of current amplitude modulation technologies in the THz range using electrical control. Modulators based on MEMS^11,12,46^ and VO_2_^13,14,47^ offer a large modulation depth combined with low insertion losses and capability of operating across a wide frequency range, but are intrinsically limited to speeds of few 10 kHz, due to the need for mechanical movement or thermal activation of the phase change in VO_2_, respectively. Another approach enabling broadband modulation with a large depth is the gating of graphene as an optically active element with an ion gel, a method which particularly stands out due to the low gate voltages required to achieve large Fermi level tuning and change in optical response^18–20^. Devices employing liquid crystals rely on a change in resonant frequency due to the dependence of the refractive index on the order within the liquid crystal, and also offer a large modulation depth with low insertion losses in a narrow band^15,16,48^. However, just as the ion gel gating, these methods are restricted to sub-few kHz modulation speeds owing to the slow motion and re-ordering of ions/liquid crystal molecules. Solid state devices that do not rely on mechanical or thermal changes promise higher speeds^38,39^, although in the case of large area unstructured graphene sheets, the RC constant associated with the large device area necessary to operate in the THz range make it challenging to break into MHz modulation speeds. The use of metamaterials allows separation of the optically active device area (determined by the radiation wavelength) and of the electrically controlled area, significantly reducing the latter. As the main RC contribution is determined by the electrically gated area, this approach enables orders of magnitude higher modulation speeds^1,34,40^, but in a narrow band, due to the LC-resonant nature of metamaterials. In the sub-THz range (0.34 THz), metamaterials based on III–V materials have shown capability of modulation with high speeds and large depth such as 1 GHz speed on AlGaN/GaN high-electron-mobility transistors (HEMTs)^22^ and reaching speeds of ~30 GHz in GaAs Schottky diode guided wave modulators^49^. But device demonstrations at higher operating frequencies show notably reduced performance, as seen from the 0.835 THz AlGaN/GaN HEMT-based device demonstrated in ref. ^37^. The downscaling of AlGaN/GaN-based metamaterials for operation at an order of magnitude higher frequency than 0.34 THz, around 2 THz, is not trivial: it will require solving issues such as an increase in Ohmic contact resistance, annealing uniformity and reliability on a (sub-)micron scale. More generally, conventional HEMT-based semiconductor designs can face limitations due to fabrication constraints on the ohmic contact size and mesa etching, which would lead to restrictions on the antenna design and, in turn, on device performance. By contrast, the choice of graphene as the 2D material allows a direct, straightforward contact to the metallic metamaterial structure, a flexible choice of substrate material on which the modulator is fabricated, and capability of applying both back gate and top gate voltages.

**Table 1:** Comparison of exemplary terahertz amplitude modulation technologies using electrical control

Device type	Modulation depth	Type (R/T)	Frequency coverage	Modulation speed	Insertion loss	Band-width	Operating voltage	Ref.
Micro-electromechanical systems (MEMS)	5.2 dB	T	0.1 to 1.5 THz	20 kHz	~3.3 dB	Broad	30 V	^11^
	16.5 dB	T	0.48 THz	<1 kHz	2 dB	Narrow	33.5 V	^12^
Vanadium oxide (VO_2_)	11 dB	T	0.7 THz	0.1 to 1 Hz	1.5 dB	Broad	2 to 12 V	^13^
	30 dB	R	0.35 to 0.76 THz	~0.1 to 1 Hz	11.8 dB	Broad	18 mA	^14^
	–	R	0.43 THz	7 kHz	–	Narrow	4 mA	^47^
Liquid ion gating of graphene	2.2 dB	R	1.5 to 5 THz	N/A, restricted by liquid ions (<few kHz expected)	2.4 to 4.4 dB	Broad	± 0.6 V	^18^
	11.5 to 20 dB	T	0.1 to 2.5 THz		5.7 to 11.4 dB	Broad	3 V	^19^
	35 dB	R	0.3 to 0.6 THz		–	Narrow	2 V	^20^
Liquid crystals	2.4 to 6.0 dB	R	3.67 THz	−(≪1 kHz)	~6.2 dB	Narrow	15 Vpp	^15^
	~20 dB	T	1.77 THz	−(≪1 kHz)	3 dB	Narrow	50 V	^16^
	~17 dB	R	0.75 THz	few Hz	~1.2 dB	Narrow	20 V	^48^
Unstructured graphene, solid state device	21.5 to 30 dB	R	0.2 to 1.6 THz	10 kHz	12 dB	Broad	±12 V	^39^
	10 dB	R	1.9 to 2.7 THz	20 kHz	7 to 11 dB	Narrow	10 V	^38^
Graphene metamaterial	0.17 dB	R	2 THz	105 MHz	–	Narrow	10 Vpp	^1^
	6.4 dB	R	0.3 to 1.4 THz	–	–	Broad	±60 V	^40^
	7.6 dB	T	0.74 THz	>3 GHz	13.5 dB	Narrow	−120 V to +25 V	^34^
GaN HEMT metamaterial	23.1 dB	T	0.34 THz	1 GHz	–	Narrow	7 V	^22^
	1.7 dB	T	0.835 THz	~20 MHz	~2.5 dB	Narrow	3 V	^37^
This work	40.1 dB	R	2.15 THz	30 MHz	~10 dB	Narrow	−80 to 180 V	
	45.7 dB	R	1.68 THz			Narrow	0 to 50 V	

The device parameters were evaluated based on data presented in the references with methods most comparable with the analysis in our work

However, one of the major challenges in achieving full modulation in graphene-based metamaterial modulators is the finite conductivity of graphene at the Dirac point^1,30,32–34^. Previous approaches aiming to overcome this inherent limitation included devices with a graphene/ionic liquid/graphene sandwich structure^19^; a quarter-wave resonance cavity^18,38^; Brewster-angle devices^39^ and reflection devices using liquid ion gating^20^. However, while up to 30–35 dB of intensity modulation was demonstrated in this way, the modulation speeds were limited to few 10 kHz.

The use of metamaterials in a reflection geometry provides more opportunities to realise a large modulation depth. Initially, thin metal films with precisely controlled thickness were used as an impedance-matching layer to zero out reflection in a static approach^50,51^; more recently, this approach has been expanded to an active tuning using GaAs Schottky-type metasurfaces in the sub-THz range^52^. Alternatively, active materials can be integrated into metamaterial perfect absorbers to enable dynamic modulation in the mid-infrared range^9^. In our work, we demonstrate zeros in reflection based on substrate-side incidence of radiation, despite the limited finite conductivity range available in graphene.

Moreover, we are able to obtain two reflection zeros at two distinct colours/frequencies in the THz range using just one gate voltage as the control parameter thanks to the tuneable capacitance approach. The operating frequencies of the tuneable capacitance modulator are determined by two key factors: the geometry of the metallic array and the size of the air gap. Stretching or compressing the unit cell of the metallic array will alter the radiative loss of the structure and hence the *Q*-factor of the resonance while maintaining the same resonant frequency. The separation between the two “colours” is governed by the air gap capacitance, which can be adjusted either by modifying the size of the air gap or by introducing another dielectric material within it.

Our approach does not rely on external cavities for modulation enhancement and employs metamaterials that have the potential for high-speed operation. The reconfiguration speed, measured on the 2.15 THz-device, is 30 MHz. This is several orders of magnitude higher than the typical speeds possible with ionic liquid gating or large-area unstructured graphene. The speed can be further increased by reducing the device size. The current device size of 1.3 mm × 1.3 mm, chosen here conservatively large by using 36 × 36 unit cells, can be scaled down to a value around *λ* × *λ* ≈ 140 µm × 140 µm by reducing the number of unit cells. The corresponding decrease of the device’s capacitance by a factor of ca. (1.3 mm/140 µm)^2^ $\approx$ 86 could push the operational speed into the GHz range. While parasitic effects such as contact resistance may play a larger role in this case, it is expected that speeds of several GHz will be achievable by transferring the presented modulation principle to top-gated device architectures^22,34,53^ to provide even larger modulation speeds and also reduce the gate voltage bias needed to drive the modulator.

In our work, the experimental results were demonstrated on two exemplary graphene-metal metamaterial terahertz modulators, both of which achieve a measured amplitude modulation in excess of 99%: 45.7 and 40.1 dB, respectively. One of them was fabricated using our own CVD-grown graphene and operates at a centre frequency of 1.68 THz, the other was fabricated using graphene from a commercial supplier and was designed for a centre frequency of 2.15 THz. The different frequency was obtained from a geometric scaling of the unit cell periods, proving the frequency scalability of our approach, while the different graphene sources showcase the robustness of the proposed modulation method.

**Fig. 7:**
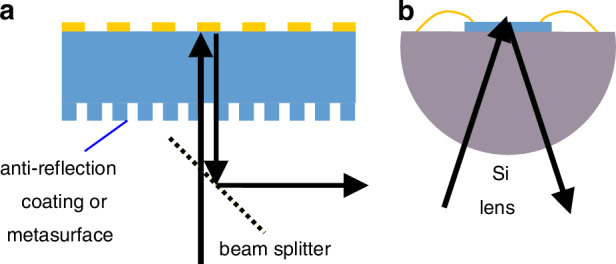
Proposed device implementations in optical systems. **a** Normal incidence reflection with anti-reflection coating or metasurface suppressing reflection from the back side; **b** coupling of a silicon lens to the device backside

The integration of the demonstrated metasurface modulator into systems for practical applications will require suppression of the reflections from the back side of the substrate. This can be realised by applying an anti-reflection coating to the back surface, or by the creation of an anti-reflection metasurface^54,55^ on the substrate side, see Fig. 7a. This minimises these reflections, enhancing impedance matching and enabling near-unity modulation depth. Using a beam-splitter, the incident and reflected beams can be separated under normal incidence on the device. Alternatively, the radiation can be coupled in using a silicon lens, Fig. 7b.

Besides using a beam-splitter, another approach to separate incident and modulated beams is to use oblique incidence. To assess the device performance at oblique angles, we also simulated p-polarised light at 10° angle of incidence in the substrate (this corresponds to ≈36° in air by Snell’s law). The results confirm that two zero-reflectance points persist within a graphene conductivity range of 0.2–0.9 mS. We note that the frequencies where the reflection vanishes shift by up to 0.1 THz, see Supplementary Information, Section 3. This highlights the resilience of the proposed modulation mechanism. Moreover, it demonstrates that the angle of incidence is an additional tuning parameter of the operational frequency of the modulator.

Some applications may require a broader operational frequency window, while a lower modulation depth is acceptable. If a modulation depth of e.g. 7.6 dB is sufficient, as in ref. ^34^, then the tuneable capacitance modulator can be used over a range of 2.025–2.417 THz, as shown in Fig. 5c, i.e. demonstrates a significantly enhanced operational window with 20% frequency tunability. This is much wider than offered by single-frequency devices, see Fig. 6c, f, and on a similar level as in ref. ^41^, where semiconductor-based resonance frequency tuning of metamaterials has been demonstrated, but required pulsed laser sources to achieve the very large conductivities needed. In contrast to previous works, the graphene conductivity required to achieve unity modulation lies in the range of 0.3–0.65 mS, which is well accessible by standard commercial CVD-grown graphene and can be electrically controlled without the need for high power optical pumping.

Future research will help to further improve the performance parameters of the proposed modulator. While we present data up to 180 V voltage on the 2.15 THz device, the 1.68 THz device achieves amplitude modulation in excess of 99% (45.7 dB) when varying the gate voltage from 0 to +50 V, and 30 dB modulation can be achieved even within a 0–20 V range. These are moderate voltage levels accessible by state-of-the-art function generators. The 1.68 THz device operates at a lower voltage because its zero in reflection (which depends on the graphene doping level and geometry-dependent *σ*(*ω*)) occurs at 0 V gate voltage. The required voltage of our devices can be further reduced by decreasing the 300 nm SiO_2_ insulating layer thickness to ~90 nm. Alternatively, the use of the wide band-gap dielectric Al_2_O_3_ deposited via atomic layer deposition can bring the Dirac point to a lower voltage, besides further reducing the required dielectric thickness. In combination with a top-gated device architecture, efficient device operation is expected with biasing in the range around or below 10 V.

Our device has an insertion loss of ~10 dB, as it was designed with focus on modulation depth, tunability, speed, and reproducibility, but not on minimising losses. Strategies such as exploiting critical coupling conditions^20^, using Salisbury screen-type modulators, or resonant cavity designs could significantly reduce insertion loss without sacrificing modulation depth. Additionally, modifying the antenna geometry—such as stretching the unit cell in the *x*-direction while compressing it in the *y*-direction—can help reduce radiative losses while maintaining the operating frequency; the resulting increase in *Q*-factor is expected to reduce the losses at resonance. Reductions in contact resistance between graphene and metal, achieved through microstructuring techniques of the graphene-metal interface^56^ could expand the tuneable range of graphene conductivity and further reduce insertion losses.

Already at its current performance level, our modulator has several possible applications as a device, particularly in cases where a sufficiently large power budget is available. It can be integrated with quantum cascade lasers (QCLs), enabling modulation of their operating point of maximum power output. As THz QCLs have already been shown to deliver output powers >1 W^57^, a loss of ~10 dB with extinction of ~60 dB would allow output power modulation in a huge dynamic range of ~1 µW to ~100 mW, with sufficient output power for many detectors used for e.g. imaging purposes. Importantly, given the very low input impedances of QCLs of a few Ohms, it is difficult to modulate them at the maximum power point by direct electronic modulation. As a modulator operating independently of the QCL laser operation, our device can modulate the QCL output without disturbing operation of the laser itself and causing inadvertent fluctuations of its operating frequency. Alternatively, by intentionally coupling radiation reflected from the modulator back into the QCL, one can exploit self-mixing phenomena within the QCL to tune the QCL output power and frequency^58^, by controlling the amount of light coupled back into the QCL. Other applications where high output powers are available include free electron laser facilities. Here, the device can be used to decimate THz pulses filtered to the desired frequency of interest, and the repetition rate of 13 MHz at e.g. FELBE makes it possible already at the current modulation speed. Finally, with an extinction of −60 dB, the device demonstrates excellent suppression of the reflectance, enabling its application as a tuneable notch filter. By changing the air gap ratio ${r}_{{\rm{air}}}$, the frequency tunability of the notch filter can be balanced against the maximum achievable extinction across the frequency band.

The demonstration of capacitive tuning via nanoscale tuneable capacitors opens up a myriad of opportunities, enabling previously demonstrated amplitude, phase, or polarisation modulator designs to be substantially improved by a simple yet effective change of the 2D electron gas design in the region of confined electric fields. In our work, we chose a brickwork antenna structure, similar to the top-gated geometries in refs. ^22,34,37^, as an example to illustrate the performance of our approach, but the design approaches we presented can be readily transferred to many other modulator geometries—including those which cannot be converted to a top-gated geometry in a straightforward way, such as split-ring resonator geometries, polarisation modulator designs, or chiral metamaterials as those in refs. ^3,33^. Similarly, the back excitation approach to achieve unity modulation can be employed in a multitude of metamaterial modulator device architectures, including semiconductors other than graphene as the active element.

In conclusion, the common approach to convert metamaterials in the terahertz range to active modulators is to shunt the capacitive gap in a unit cell with a 2D electron system such as graphene, which serves as a gate voltage-tuneable resistor and influences the transmission or reflection spectra of the device. While functioning, the damping of the resonance by the predominantly resistive loading of the structure is an inefficient use of the 2D material, and in the case of graphene, the achievable modulation depth is also compromised by the finite conductivity at the Dirac point, which does not allow it to become fully depleted, in contrast to conventional semiconductors.

Here, we proposed and experimentally demonstrated a tuneable capacitance modulator device design by exploiting graphene not as a variable resistor shunting the LC circuits of the metamaterial structure, but as a tuneable nanoscale lateral capacitor, that modifies the resonance frequency. In response to the issue of finite graphene conductivity, we used a reflection modulator design operating in back-side excitation, which exploiting destructive interference of the Fresnel reflection components to enhance the modulation depth.

To the best of our knowledge, our tuneable capacitance metasurface modulator demonstrates the highest experimentally measured modulation depth to date for a non-integrated graphene-based metamaterial (40.1 and 45.7 dB on two different devices), while maintaining a device architecture compatible with high-speed modulation, as evidenced by the 30 MHz measured reconfiguration speed. Our results open up new opportunities in the area of communication technologies, real-time imaging, and analogue computing with optical waveforms in the terahertz range.

## Materials and methods

### Sample fabrication

The samples are fabricated on a 525 ± 25 μm boron p-doped high resistive silicon substrate (>100 Ωcm resistivity) with a double-sided coating of a 300 nm SiO_2_ layer. The back-side coating is removed by reactive ion etching, leaving the SiO_2_ layer only on the top side. The graphene used is monolayer graphene on copper, grown by chemical vapour deposition. For the device operating at 2.15 THz, it was procured from a commercial supplier (Graphenea); for the device operating at 1.68 THz it was grown in-house in our own CVD system^59,60^. The graphene is transferred using a wet transfer technique onto the sample. The graphene patches are created using electron beam lithography and subsequent selective etching of the graphene in an oxygen plasma. The titanium–gold metal layer of the brickwork arrays (14 nm Ti, 156 nm Au) is thermally evaporated after a second electron beam lithography step. See Supplementary Section SI-1 for more details.

### TDS measurements

The experimental set-up used is Tera K15 from Menlo Systems. Measurements are conducted at room temperature in a dehumidified dry air environment (150 ppm by volume, at the same impurity level as commercial gaseous nitrogen). To extract the reflectance, the first and second peaks are selected using a Blackman–Harris window function with a 12 ps full width. The reflectance is then calculated from the ratio of the squared absolute values of the Fourier transforms of the second and first transmitted peaks, *P*_2_ and *P*_1_. As seen from Fig. 4f, their ratio equals

7 $$\frac{{P}_{2}}{{P}_{1}}=\frac{{{T}_{{\rm{D}}}{T}_{{\rm{S}}}R}_{{\rm{S}}}{R}_{{\rm{D}}}}{{T}_{{\rm{D}}}{T}_{{\rm{S}}}}={R}_{{\rm{S}}}{R}_{{\rm{D}}}$$

where ${R}_{{\rm{D}}}$ is the reflectance when the device is excited from the back side (substrate side), and ${R}_{{\rm{S}}}$ is the reflectance of the substrate and air interface. To obtain the reflectance of the device ${R}_{{\rm{D}}}$, this value is divided by the reflectance of the silicon substrate *R*_S_ = [(*n*_Si_ − 1)/(*n*_Si_ + 1)]^2^ = 0.300, with *n*_Si_ = 3.425^61–63^. We note that due to the use of high-resistivity wafers, we can neglect in Eq. (7) the absorption of the radiation within the substrate, which we estimated from substrate transmittance data to be no more than a few percent.

### Speed measurement

We define the modulation speed as the sine-wave modulation frequency at which the modulation response has fallen off to half of its value at DC/low frequencies. The measurement speed of our THz time-domain system is significantly lower than the response time of the device. To characterise the speed, we make use of an established measurement method^1,33^ that relies on the fact that the temporal average of the transmission under the presence of an AC sine wave differs from the transmission without any applied AC signal. This change of the averaged transmitted signal with and without AC signal can be measured by applying an AC sine wave of varying frequency to the device, even if the speed of the detector response is much smaller than the modulator speed. We use a TG5011A function generator to apply a 19.8 V peak-to-peak AC sine wave between back gate and metamaterial modulator contacts at zero DC bias. The transmitted electric fields are measured using TDS with and without the AC signal. The difference is evaluated by integrating the ratio of the transmission data with and without AC voltage applied over a frequency range from 1.9 to 2.1 THz, where the maximum difference between the two cases is observed. This normalised difference Δ*T* is shown in Fig. 5d. The error bars represent the standard deviation from six measurements.

## Supplementary information

Supplementary Information

## Data Availability

The underlying data are deposited at the University of Cambridge Apollo repository^64^ under 10.17863/CAM.116475.
