# Supplementary material for: Achieving 100% amplitude modulation depth in the terahertz range with graphene-based tuneable capacitance metamaterials
Source: Light Sci Appl. 2025 Aug 4;14:256. doi: 10.1038/s41377-025-01945-4 (PMC12319094; doi:10.1038/s41377-025-01945-4)
Supplement: Supplementary file 1 — Supplementary Information [file 41377_2025_1945_MOESM1_ESM.pdf]

Supplementary Information to:

Achieving 100% Amplitude Modulation Depth in the Terahertz Range with Graphene-Based Tuneable Capacitance Metamaterials

Ruqiao Xia[a,1], Nikita W. Almond[a], Wadood Tadbier[b], Stephen J. Kindness[a], Riccardo Degl'Innocenti[c], Yuezhen Lu[d], Abbie Lowe[a], Ben Ramsay[a], Lukas A. Jakob[a], James Dann[a], Stephan Hofmann[b], Harvey E. Beere[a], Sergey A. Mikhailov[e], David A. Ritchie[a,f], Wladislaw Michailow[a,2]

[a:] Cavendish Laboratory, University of Cambridge, CB3 0HE Cambridge, UK

[b:] Department of Engineering, University of Cambridge, Cambridge, UK

[c:] School of Electronic Engineering and Computer Science, Queen Mary University of London, Mile End Road, London E1 4NS, UK

[d:] Department of Electronic & Electrical Engineering, Faculty of Engineering Sciences, University College London, London, WC1E 7JE, UK

[e:] Institute of Physics, University of Augsburg, D-86135 Augsburg, Germany

[f:] Centre for Integrative Semiconductor Materials and Department of Physics, Swansea University, Bay Campus, Fabian Way, Swansea SA1 8EN, UK

Electronic mails: 1 - rx224@cam.ac.uk; 2 - wm297@cam.ac.uk

SI-1: Fabrication of the modulator devices

The graphene-based modulator is fabricated on a 4-inch, 525 ± 25 μm thick boron-doped silicon substrate (≥ 100 Ω·cm) with a 300 nm SiO_2 top coating. The SiO_2 layer on the back side is removed via hydrogen fluoride etching or via dry inductively coupled plasma reactive ion etching, ensuring that the SiO_2 layer remains only on the top side. The device structure consists of three layers: the alignment marker layer, the graphene layer, and the metal layer. The features in each layer are defined and patterned using electron beam lithography (EBL), using a Leica VB6-UHR operating at 100 kV accelerating voltage. Proximity Effect Correction for each layer of the pattern was performed using GenISys TRACER and BEAMER software.

1. Alignment Marker Layer

A7 495k polymethyl methacrylate (PMMA) resist is spin-coated at 4000 rpm and baked at 180°C for 10 minutes. The EBL patterning used a 10 nm beam step, a 41.5 nA beam current, and a dose to clear of 620 μC cm^{-2}. After development in a mixture of IPA (isopropanol): MIBK (methyl isobutyl ketone) : MEK (methyl ethylketone) (15 : 5 : 1) for 8 seconds, a 10/30 nm Ti/Au layer is thermally evaporated. The subsequent lift-off process in acetone (and IPA rinse) defines the alignment markers. The wafer is then diced into 10 mm × 12 mm chips.

2. Graphene Layer

The samples presented used graphene obtained from two suppliers.

The modulator presented in Fig. 5 made use of graphene purchased from Graphenea, a sample of monolayer graphene on Cu with PMMA Coating.

The modulators presented in Fig. 6 made use of graphene that we fabricated via chemical vapour deposition as follows [59, 60]:

A 25 μm 99.8% Cu foil (Alfar Aesar) was cut down to 6 cm × 6 cm sheets, sonicated in acetone (99.9% analytical grade), followed by sonication in isopropyl alcohol (IPA, 99.9%), and dried with nitrogen. The foils were then oxidized at 200 °C in a preheated, atmospheric oven for a period of 2 hours leading to a uniform Cu oxide of approximately 200 nm thickness. All CVD graphene growth experiments are performed in a commercial Aixtron Black Magic Pro 4-inch cold wall system with a base pressure of approximately 0.05 mbar. The total pressure for all heating, annealing, growth, and cooling stages is 50 mbar. The CVD process starts with ramping up in Ar to a temperature of around 1065 °C, achieved within 20 min. The foils are then annealed in Ar for 30 minutes. Oxide reduction was then performed using a mixture of hydrogen (H_2) and argon (Ar) gases at flow rates of 100 sccm and 500 sccm, respectively, for 2 hours. Graphene growth occurs during an exposure stage of length growth of 60 minutes in CH_4, H_2, and Ar (flow rates ratio 0.32:64:576 sccm, respectively). The samples were cooled in Ar to <200 °C in approximately 1 hour.

In both cases, the graphene is then transferred onto the SiO2/Si substrate using a PMMA scaffold. First, the copper substrate supporting the graphene is etched using a 0.2 mol L^{-1} ammonium persulfate (APS) solution, and the floating graphene/PMMA is cleaned in deionized (DI) water before being transferred onto the target substrate. Gradual annealing from 40°C to 150°C improves adhesion. The PMMA is removed with acetone.

The graphene is patterned using EBL, with a 5 nm beam step, a 3.0 nA beam current, and a dose to clear of 135 μC cm^{-2}, with ma-N 2400 series negative resist, developed with ma-D 525 for 30 seconds and unwanted graphene is then removed by oxygen plasma ashing at 100 W for 2-3 minutes. Thereafter, acetone and IPA rinse are used to remove the ma-N resist etch mask, leaving the patterned graphene layer intact.

3. Metal Layer

A double-layer positive e-beam resist consisting of PMMA A7 followed by 950k PMMA A11, diluted 1:5 with MIBK, is spin-coated at 4000 rpm and baked at 180°C for 10 minutes. The EBL patterning used a dose to clear of 620 μC cm^{-2}. Fine features were patterned using a 5 nm beam step and 21.7 nA beam current, whereas coarse features (e.g. bond pads) used a 20 nm beam step and 80 nA beam current. After development in a mixture of IPA : MIBK : MEK (15 : 5 : 1) for 8 seconds, a 14/156 nm Ti/Au layer is then deposited via thermal evaporation, and the unwanted metal is lifted off in acetone (and isopropanol rinse), completing the modulator fabrication.

SI-2: Transmittance data

The TDS measurements of the transmittance of the modulators are presented in this section. The transmittance data is evaluated from the 1st transmitted peak, referenced against the transmittance without sample, and includes the loss at the first air-silicon interface. All three

modulators have an active device area of 1.3 mm by 1.3 mm. In summary, the three devices demonstrate an intensity modulation depth of 31–37% in transmission.

Figure S1 shows the transmittance of the THz-TDS measurements of the device presented in Fig. 5 under gate voltages ranging from –160 to +180 V. The transmittance measurements of the device demonstrate a modulation depth of 2.04 dB at 2.15 THz, corresponding to 37.5 % modulation depth in intensity.

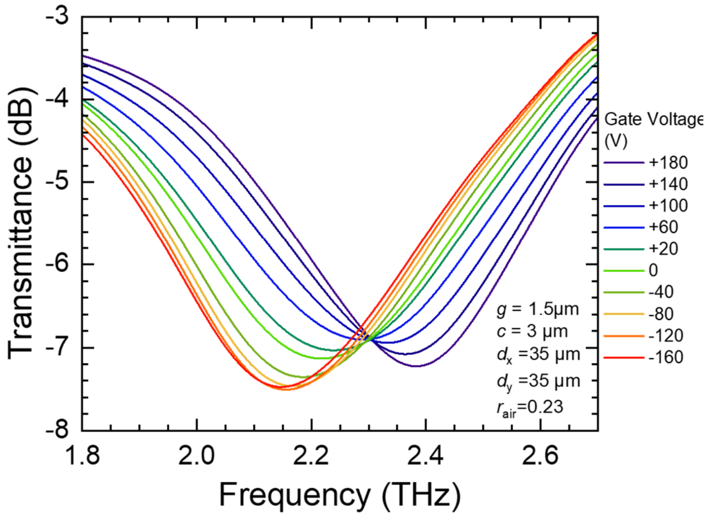

Figure S1: Transmittance results for the tuneable capacitance modulator (with air gap, r_{air} = 0.23). TDS measurements with gate voltage varied from -160V to 180V of the brickwork antenna tuneable capacitance metamaterial as a function of frequency. The device structure has the parameters g = 1.5 μm, c = 3 μm, $\boldsymbol{d_x}$ **= 35 μm,** $\boldsymbol{d_y}$ **= 35 μm**. The device is fabricated on a sample with an active device area of 1.3 mm by 1.3 mm, comprising 36 × 36 unit cells and using graphene from a commercial supplier.

The two devices presented in Fig. 6 have identical metallic structure designs but different active element configurations: one with an air gap and one without. The brickwork modulators are fabricated on a sample with an active device area of 1.3 mm by 1.3 mm, comprising 28 × 28 unit cells. The measurements of the transmittance for both devices are plotted using the same scale on the x and y axes, enabling direct comparison of the data. The tuneable capacitance modulator with air gap in the graphene patches achieves 35 % intensity modulation depth in transmittance at a centre frequency of 1.68 THz when varying the gate voltage from 0 V to +50 V. By contrast, the transmittance data for the no air-gap, variable-resistor device shows a modulation depth of 31 % when varying the gate voltage from +50 V to –100 V, see Fig. 3. In the latter device, there is no obvious shift in resonance frequency across different gate voltages.

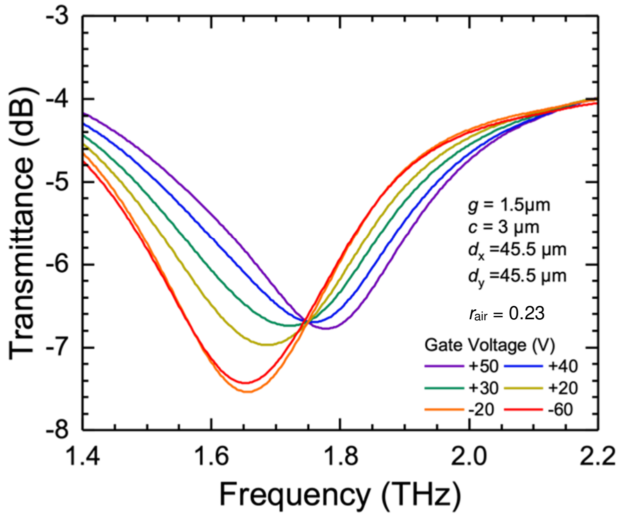

Figure S2: Transmittance results for the tuneable capacitance modulator (with air gap, r_{air} = 0.23). The device structure has the parameters g = 1.5 μm, c = 3 μm, $\boldsymbol{d_x}$ **= 45.5 μm,** $\boldsymbol{d_y}$ **= 45.5 μm.** TDS measurements with gate voltage varied from –60V to +50V of the brickwork antenna tuneable capacitance metamaterial as a function of frequency.

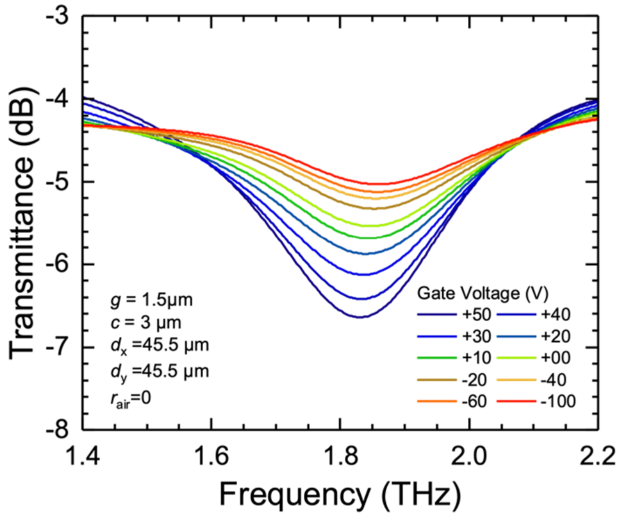

Figure S3: Transmittance results for the variable resistance modulator (without air gap). TDS measurements with gate voltage varied from –100V to +50V of the modulator as a function of frequency. The device structure has the parameters g = 1.5 μm, c = 3 μm, $\boldsymbol{d_x}$ **= 45.5 μm,** $\boldsymbol{d_y}$ **= 45.5 μm.**

SI-3: Modulation performance under oblique incidences

In this section, we present the numerical simulations of the reflectance of the metasurface presented in Fig. 5 when excited by p-polarized light at varying angles of incidence. The simulated structure has the parameters g = 1.5 μm, c = 3 μm, d_x = 35 μm, d_y = 35 μm, and $r_{air} = 0.23$. The angle of incidence is defined within the substrate. A detailed analysis of how

the angle of incidence influences the device's optical properties is provided, highlighting key trends and performance variations.

Figures S4 and S5 illustrate the simulated reflectance of the device under different angles of incidence. Specifically, Figure S4 presents the reflectance for a 5° angle of incidence within the substrate, which corresponds to approximately 17° in air using Snell's law, if considering the refraction at the air-substrate backside interface. Figure S5 shows the reflectance for a 10° angle of incidence in the substrate, equivalent to 36° in air. The results show that the device maintains two deep resonance dips within the graphene conductivity range under these angles of indicence. This confirms that the designed structure supports the presence of two zero-reflectance points, even under oblique angles of incidence.

However, the optical response exhibits slight variations with changing incident angles:

- The overall resonance shifts toward lower frequencies, with a frequency shift of less than 0.1 THz observed between Figures 4 and 5.
- The conductivity values at which reflectance is minimized differ between incident angles. For the 5° case, the zero-reflectance points occur at conductivity values of approximately 0.30 and 0.85 mS. For the 10° angle of incidence, these values shift to around 0.25 and 0.90 mS.

These observations highlight the angular dependence of the device's optical response while maintaining its fundamental resonance characteristics.

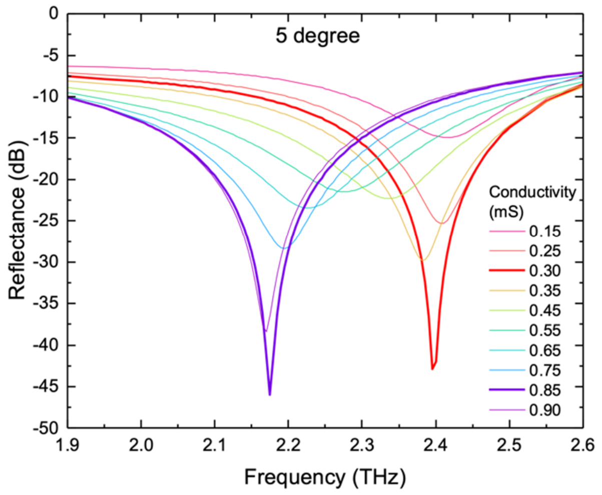

Figure S4: Simulated reflectance at 5 degrees angle of incidence of a tuneable capacitance metamaterial. The simulated structure has the parameters g = 1.5 μm, c = 3 μm, d_x = 35 μm, d_y = 35 μm, and $r_{air} = 0.23$.

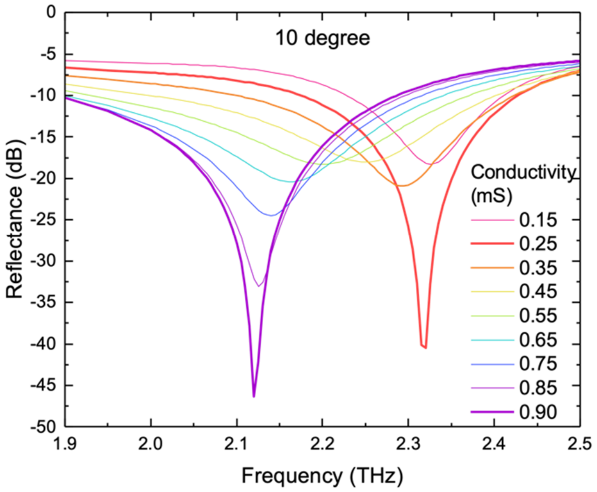

Figure S5: Simulated reflectance at 10 degrees angle of incidence of a tuneable capacitance metamaterial. The simulated structure has the parameters g = 1.5 μm, c = 3 μm, d_x = 35 μm, d_y = 35 μm, and $r_{air} = 0.23$.
